# Alpha_2_‐adrenoceptor agonists inhibit form‐deprivation myopia in the chick

**DOI:** 10.1111/cxo.12871

**Published:** 2019-01-30

**Authors:** Brittany J Carr, Cynthia T Nguyen, William K Stell

**Affiliations:** ^1^ Department of Neuroscience, Cumming School of Medicine University of Calgary Calgary Alberta, Canada; ^2^ O'Brien Centre for the Bachelor of Health Sciences, Cumming School of Medicine University of Calgary Calgary Alberta, Canada; ^3^ Department of Cell Biology and Anatomy, Cumming School of Medicine University of Calgary Calgary Alberta, Canada

**Keywords:** adrenoceptor, agonism, atropine, brimonidine, myopia

## Abstract

**Background:**

The putative myopia‐controlling receptor is thought to be muscarinic acetylcholine receptor subtype M_4_, because mamba toxin‐3 can inhibit form‐deprivation myopia in chicks at a far lower concentration than atropine. However, mamba toxin‐3 is equally potent at the human α_1A_‐, α_1D_‐, and α_2A_‐adrenoceptors. To test the hypothesis that α‐adrenoceptors might be involved in regulation of eye growth, the treatment effects of α_2_‐adrenoceptor agonists brimonidine, clonidine, and guanfacine, and antagonist yohimbine, on form‐deprivation myopia in the chick were measured.

**Methods:**

Right eyes of White Leghorn chicks were goggled with diffusers to induce form‐deprivation myopia; left eyes were left open as controls. Goggled eyes were injected intravitreally with 20 μL of vehicle, or 2, 20, or 200 nmol of brimonidine, clonidine, guanfacine, or yohimbine, 24, 72, and 120 hours after goggle application. Alternatively, myopia was inhibited physiologically by goggle removal for two hours, and the α_2_‐adrenoceptor antagonist, yohimbine, was injected to test whether it could block this type of myopia inhibition. One day after the last injection, refractive error and axial length were measured.

**Results:**

Brimonidine (20 and 200 nmol) and clonidine (200 nmol) effectively inhibited experimentally induced increases in negative refractive error and axial elongation. All doses of guanfacine significantly inhibited induced negative refractive error, but only 20 and 200 nmol significantly inhibited axial elongation. Yohimbine had no effect on form‐deprivation myopia, but 200 nmol reduced the myopia‐inhibiting effect of goggle removal.

**Conclusion:**

High concentrations of α_2_‐adrenoceptor agonists, similar to those required by atropine, inhibited chick form‐deprivation myopia; antagonism by yohimbine had no effect. High‐concentration yohimbine partially interfered with emmetropisation in form‐deprived chicks experiencing normal vision for two hours per day. These data support the hypothesis that treatment with high concentrations of adrenergic drugs can affect experimentally induced myopia and normal visual processes.

Myopia is a refractive disorder characterised by the inability to see distant objects clearly. Untreated myopia is the most common childhood vision disorder and the leading cause of adult visual impairment worldwide. There is no cure, and no universally accepted pharmaceutical therapy to slow myopia progression. Some success has been found with atropine, a muscarinic acetylcholine receptor (mAChR) antagonist. Treatment with 0.01 per cent atropine has become fairly common in North America and Southeast Asia,^1^, ^2^ but the ocular signalling cascades modulated by atropine remain poorly understood.

Research in this area has been biased toward the assumption that, because atropine and a few other mAChR antagonists inhibit myopia when applied at high concentrations, mAChRs must regulate myopia development. This has not been conclusively proven, however, and many questions remain about the mechanism(s) underlying childhood myopia, experimentally induced myopia in animals, and the target receptors and pathways responsible for inhibition of eye growth.

Currently, the M_4_ mAChR subtype has been named the receptor most likely to mediate myopia inhibition by atropine. This is because mamba toxin‐3 (MT3), a component of the venom of the East‐African green mamba (*Dendroaspis angusticeps*), is selective for mAChR M_4_ over all other mAChR subtypes in mammals,^3^ and it inhibits myopia in chicks and tree shrews at a much lower concentration than required by atropine (intravitreal injections of 10 μL of 2.5–10 μmol/L versus 20 μL of 10–100 mmol/L, respectively).^4^, ^5^

While it is true that MT3 has a high inhibitory potency at human mAChR M_4_, it has equally high potency at α_1A_‐, α_1D_‐, and α_2A_‐adrenoceptors (1–10 nmol/L), moderately high potency at α_1B_‐ and α_2C_‐adrenoceptors (27–50 nmol/L), and low potency at mAChR M_1_ (200 nmol/L).^6^ Therefore, at the concentrations used to inhibit form‐deprivation myopia, any one of these α‐adrenoceptors, as well as mAChR M_1_, could be the ‘true’ mediator of myopia inhibition by MT3.

The most common method for testing whether a drug is involved in eye growth is intravitreal injection of usually very high concentrations of the drug into the eye of an animal undergoing experimentally induced myopia – most commonly a chick. An important consequence of applying high concentrations of drugs is the increased risk of binding to off‐target receptors, as no drug is perfectly selective for a single receptor. For example, the three most effective myopia‐inhibiting mAChR antagonists – atropine, himbacine, and MT3 – bind to human α_2A_‐adrenoceptors expressed in HEK293T cells when applied at concentrations at or above 45 μmol/L, 17 μmol/L, and 15 nmol/L, respectively.^7^

Off‐target binding of drugs is not a well‐studied line of inquiry, but there is interesting evidence from the chick form‐deprivation model that supports a role for off‐target, and not muscarinic, receptor effects for myopia treatment with high concentrations of atropine, himbacine, and MT3. Atropine and himbacine are very potent inhibitors of the chick mAChR M_4_ receptor (710 pmol/L and 6 nmol/L, respectively), yet a high concentration of these drugs is required to achieve full inhibition of form‐deprivation myopia in the chick.

Approximating the chick eye as a sphere of a radius 4.6 mm (calculated from all control eyes in this experiment) and assuming equal distribution of the drug throughout the sphere, concentrations of injected drug expected to reach intraocular receptors would be 500 μmol/L–5 mmol/L atropine,^5^ 50–100 μmol/L himbacine,^8^ and 50–200 nmol/L MT3.^4^ Interestingly, although MT3 is the most effective drug at inhibiting form‐deprivation myopia in the chick, it is the least potent mAChR antagonist at the chick M_4_ receptor (450 nmol/L).^7^ Thus, if atropine and himbacine were inhibiting form‐deprivation myopia via the chick M_4_ receptor, the concentrations of intravitreal injections required should at least match, and in fact be much lower, than those required by MT3. This is clearly not the case. In fact, the high potency of MT3 for the α_2A_‐adrenoceptor (2–15 nmol/L) correlates better with inhibition of form‐deprivation myopia in chicks than does mAChR antagonism.^7^ It is possible then, that MT3 is much more effective against form‐deprivation myopia than atropine because it is acting upon an α‐adrenoceptor, and not mAChR M_4_.

The evidence against a role of mAChR M_4_ and for a possible role of α‐adrenoceptors in regulation of eye development encourages further investigation. To this end, the effects of various α_2_‐adrenoceptor agonists (clonidine, guanfacine, and brimonidine) and the α_2_‐adrenoceptor antagonist yohimbine, on form‐deprivation myopia in the chick were tested. It was hypothesised that yohimbine should inhibit form‐deprivation myopia, and the agonists, brimonidine, clonidine, and guanfacine, should either exacerbate it or have no effect; the opposite was found to be true. A preliminary report of these findings was presented previously (Carr BJ and Stell WK. IOVS 2016; 57: ARVO E‐Abstract 4738).

## Methods

### Ethics statements and animal housing

Animal use protocols were approved by the Health Sciences Animal Care Committee of the University of Calgary, and carried out in accordance with the Canadian Council on Animal Care and the ARVO Statement for the Use of Animals in Ophthalmic and Vision Research.

White Leghorn cockerels (Shaver and Lohmann strains) were purchased from Rochester Hatchery (Westlock, Alberta, Canada) or Clark's Poultry (Brandon, Manitoba, Canada) and delivered on post‐hatching day one (P1). Chicks were housed at the University of Calgary Health Sciences Animal Resource Centre at 26°C, on a 12:12 light‐dark schedule (lights on at 06:00 hours) and given food and water *ad libitum*. Mean illuminance in the housing and lab areas was 350–500 lux, provided by conventional indoor fluorescent lighting.

### Induction of form‐deprivation myopia and intravitreal injections

Form‐deprivation myopia was induced, starting on day P7 or P8, by affixing a translucent diffuser goggle over the right eye using contact cement; the left eye remained ungoggled, as a within‐animal control. Goggles remained in place during injections; small triangular vents were cut in the top of the goggles to facilitate needle access and promote air circulation, without significantly diminishing the form‐deprivation effect.

Prior to injections, chicks were anaesthetised with 1.5 per cent isofluorane in 50:50 O_2_:N_2_O. Once chicks were fully unconscious, 20 μL of solution was injected into the dorsal quadrant of the eye using a 26‐gauge needle attached to a 25 μL Hamilton Gastight syringe; dedicated syringes and needles were used for each treatment group and rinsed in 70 per cent ethanol between injections.

Intravitreal injections were performed at the same time each day (12:00–14:00 hours) to avoid variable interactions between injections and circadian influences on eye growth – and only every second day (24, 72, and 120 hours post‐goggling) to minimise the discomfort in the chick and any growth‐retarding effects of frequent needle‐puncture. For brimonidine treatment, only two intravitreal injections (24 and 72 hours post‐injection) were performed.

### Goggle removal and recovery from form‐deprivation myopia

For goggle removal and recovery experiments, goggles were applied using Velcro rings. Chicks undergoing yohimbine injection + goggle removal were treated on the same schedule as those undergoing form‐deprivation, but the goggles were removed immediately before each yohimbine injection (after anaesthesia) and then left off for two hours. During this time, chicks were kept in large clear bins with access to food and water and exposed to normal lab lighting (350–500 lux).

After two hours, the diffuser goggles were replaced, and the chicks were then returned to animal housing. To verify that inhibition of eye growth by α‐adrenoceptor agonists was not due to toxic effects on visual circuitry, goggles were removed from some chicks one day after completion of regularly scheduled drug injections and then those chicks were raised for an additional week with normal vision under regular lighting conditions. Chicks still grow quickly at three weeks of age, and eyes with intact retinal circuitry will correct for refractive errors induced by drug treatments or goggle‐wear by attempting to achieve emmetropisation, resulting in a reduction of the within‐animal intraocular difference.

If retinal circuitry is damaged by drug toxicity, the eyes will not emmetropise and the within‐animal intraocular difference will remain significantly large. Guanfacine was chosen for recovery, as it had the behavioural profile most similar to that of atropine and was the most effective at the lowest doses among the three agonists.

### Drugs for intravitreal injections

Drugs were dissolved in sterile phosphate‐buffered saline (PBS; Gibco 14190‐144; ThermoFisher Scientific, Waltham, Massachusetts, USA) at room temperature; yohimbine and guanfacine required gentle heating to dissolve fully at the highest concentration. Solutions were made fresh for each set of injections. The concentrations tested were within a log dose range, based on the concentration of atropine required to achieve significant inhibition of form‐deprivation myopia in these particular chick strains.^9^

### Rationale for drug selection

Brimonidine (UK 14,304; Sigma‐Aldrich, St. Louis, Missouri, USA), clonidine, and guanfacine are all α_2_‐adrenoceptor agonists with a moderate selectivity for the α_2A_‐adrenoceptor. This class of drugs was chosen because previous work demonstrated that myopia‐inhibiting muscarinic receptor antagonists can bind to α_2A_‐adrenoceptors with low potency.^7^

Brimonidine was chosen because its affinity in tissues has been reported (19 nmol/L). Previous studies have demonstrated that it can modulate chick Müller cell activity and retinal neuroprotective pathways^10^, ^11^, ^12^ and it has recently been reported to inhibit lens‐induced myopia in a small pilot study utilising guinea pigs.^13^ Furthermore, brimonidine is used commonly as a topical treatment for human glaucoma, and thus should be easily translatable to human clinical studies for protection against myopia, should it prove to be effective.

Clonidine was chosen because it is one of the most common α_2_‐adrenoceptor agonists used experimentally; its affinity has been reported in chicks (4 nmol/L)^14^ and there is precedent for neuroprotective and tyrosine hydroxylase‐regulating effects of clonidine in the rat retina.^15^, ^16^, ^17^ Tyrosine hydroxylase is the rate‐limiting enzyme in production of dopamine, which is well established as an important regulatory molecule during eye development.^18^

Guanfacine was chosen because it is somewhat selective for the α_2_‐adrenoceptor and has also been used experimentally in chicks;^19^ affinity data for guanfacine in avian tissues have not been published.

Yohimbine was chosen as the antagonist because it is commonly used, data on its affinity in avian receptors have been published (630 pmol/L),^20^ and it is selective for the α_2_‐adrenoceptor subtype.

### Measurements

One day after the final injection, goggles were removed, and refractive error (±0.50 D) was measured without cycloplegia using a streak retinoscope (Model 18100; Welch Allyn, Mississauga, Ontario, Canada) and trial lenses; working distance was approximately 0.5 m, and no correction was made for distance or the small‐eye artefact.

Subsequently, chicks were euthanised by intraperitoneal injection of 240 mg/ml Euthanyl (pentobarbital sodium; CDMV, Saint‐Hyacinthe, Montreal, Canada), followed by decapitation. Eyes were removed, extraocular tissues were dissected away, and then the globe was placed in a Petri dish supported by a PBS‐dampened paper towel for viewing perpendicular to the optic axis.

Axial length was defined as the distance from the front of the cornea to the back of the sclera. Measurements (±0.01 mm) were made with digital calipers (Model 58‐6800‐4; Mastercraft); recorded values for axial length were the average of three separate measurements.

### Data analysis

Drug treatments were found not to affect the measured parameters of control eyes (Figure S1); therefore, the treatment effects are expressed as the mean difference between values for the experimental eye (goggled, drug‐injected) and control eye (open, vehicle‐injected) ± standard deviation (SD). Statistical analysis on normally distributed data was performed using one‐way analysis of variance with Tukey's post hoc test (Prism V6.02; GraphPad Software, Inc., La Jolla, California, USA) unless specified otherwise; differences between mean values in different treatment groups were deemed significant at p < 0.05.

## Results

Absolute values for the differences in refractive error and axial length and the statistical p‐value data for all treatment groups are summarised in Table 1.

**Table 1:** Summarised data for all experiments performed

	PBS (n)	2 nmol (n, p‐value vs. PBS)	20 nmol(n, p‐value vs. PBS)	200 nmol(n, p‐value vs. PBS)
Brimonidine (form‐deprivation)				
dRE	−6.1 ± 5.2 D	−6.6 ± 4.0 D	**−2.1 ± 2.7 D**	**−0.8 ± 1.2 D**
	(14)	(17) p = 0.9768	**(17) p = 0.0161**	**(16) p = 0.0008**
dAL	0.39 ± 0.26 mm	0.38 ± 0.32 mm	**0.11 ± 0.17 mm**	**0.02 ± 0.21 mm**
	(17)	(18) p = 0.9992	**(18) p = 0.0088**	**(17) p = 0.0004**
Clonidine (form‐deprivation)				
dRE	−12.0 ± 3.8 D	−10.7 ± 2.8 D	−9.2 ± 2.0 D	**−2.2 ± 3.4 D**
	(10)	(10) p = 0.7475	(12) p = 0.1895	**(10) p < 0.0001**
dAL	0.43 ± 0.19 mm	0.31 ± 0.21 mm	0.34 ± 0.21 mm	**0.002 ± 0.24 mm**
	(10)	(12) p = 0.5178	(10) p = 0.7847	**(10) p = 0.0004**
Guanfacine (form‐deprivation)				
dRE	−16.5 ± 1.7 D	**−9.8 ± 5.2 D**	**−8.6 ± 2.9 D**	**−8.2 ± 3.0 D**
	(10)	**(11) p = 0.0004**	**(12) p < 0.0001**	**(12) p < 0.0001**
dAL	0.71 ± 0.16 mm	0.55 ± 0.27 mm	**0.43 ± 0.16 mm**	**0.34 ± 0.20 mm**
	(10)	(11) p = 0.3063	**(11) p = 0.0175**	**(12) p = 0.0007**
Guanfacine (emmetropisation)				
dRE	−0.5 ± 0.8 D	−0.8 ± 0.9 D	−0.4 ± 0.8 D	−0.4 ± 0.7 D
	(16)	(16) p = 0.8351	(14) p = 0.9440	(17) p = 0.9431
dAL	0.25 ± 0.23 mm	0.17 ± 0.22 mm	0.16 ± 0.22 mm	0.31 ± 0.34 mm
	(17)	(18) p = 0.7632	(17) p = 0.7091	(17) p = 0.9282
Yohimbine (form‐deprivation)				
dRE	−13.9 ± 4.7 D	−12.3 ± 5.9 D	−13.1 ± 4.0 D	−13.3 ± 4.0 D
	(17)	(19) p = 0.7537	(19) p = 0.9523	(18) p = 0.9837
dAL	0.58 ± 0.20 mm	0.36 ± 0.29 mm	0.41 ± 0.24 mm	0.47 ± 0.15 mm
	(17)	(20) p = 0.0547	(21) p = 0.1102	(19) p = 0.5291

Yohimbine (goggle removal)	PBS removal (n)	PBS no removal (n)	2 nmol (n, p‐value vs. PBS removal)	20 nmol (n, p‐value vs. PBS removal)	200 nmol (n, p‐value vs. PBS removal)
dRE	−4.8 ± 1.7 D	**−9.6 ± 5.7 D**	−7.0 ± 3.5 D	−6.8 ± 2.6 D	**−8.7 ± 3.1 D**
	(19)	**(22) p = 0.0008**	(19) p = 0.3371	(15) p = 0.5315	**(15) p = 0.0239**
dAL	0.29 ± 0.20 mm	**0.56 ± 0.24 mm**	0.40 ± 0.25 mm	0.32 ± 0.23 mm	0.46 ± 0.27 mm
	(20)	**(22) p = 0.0054**	(19) p = 0.6645	(15) p = 0.9973	(18) p = 0.1990

Refractive error and axial length data are represented as the intraocular differences between experimental eyes (goggled, drug‐injected eye) minus the control eyes (open, vehicle‐injected eye) ± SD. The number of animals (brackets) and statistical p‐values in comparison to control (one‐way analysis of variance + Tukey's post hoc test; p < 0.05 in bold) are listed below treatment outcomes. dAL: interocular difference in axial length, dRE: interocular difference in refractive error, PBS: phosphate buffered saline.

### Effect of α‐adrenoceptor agonists on form‐deprivation myopia and recovery

**Figure 1:**
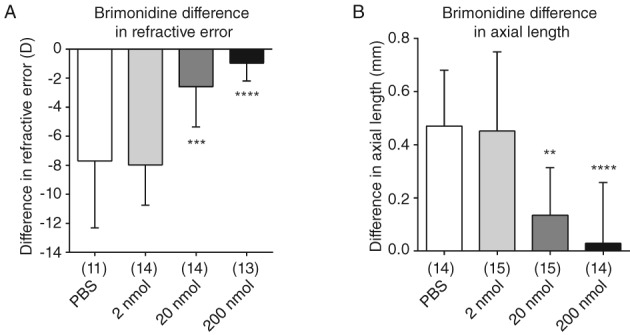
Effect of intravitreal brimonidine on form‐deprivation myopia in chicks. Twenty and 200 nmol brimonidine significantly inhibited the induced difference in A: refractive error and B: axial length compared to phosphate‐buffered saline (PBS) controls. Data are presented as the means of values ± SD. Statistics: ^****^p < 0.0001, ^***^p < 0.001, ^**^p < 0.01, ^*^p < 0.05; one‐way analysis of variance + Tukey's post hoc test. Sample sizes (n) are denoted in brackets below each column.

Intravitreal brimonidine at 20 and 200 nmol significantly inhibited the increases in negative refractive error and axial elongation induced by form‐deprivation, compared to those in PBS‐only controls; 2 nmol had no effect (n = 14–18; Table 1, Figure 1).

**Figure 2:**
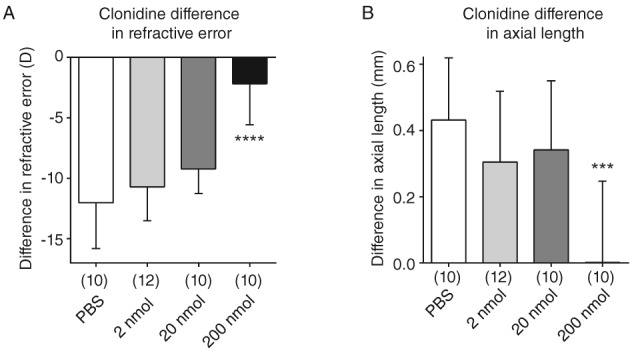
Effect of intravitreal clonidine on form‐deprivation myopia in chicks. Two hundred nanomoles clonidine significantly inhibited the induced difference in A: refractive error and B: axial length compared to phosphate‐buffered saline (PBS) controls. There was no significant effect of 2 or 20 nmol clonidine treatment. Data are presented as the means of values ± SD. Statistics: ^****^p < 0.0001, ^***^p < 0.001; one‐way analysis of variance + Tukey's post hoc test. Sample sizes (n) are denoted in brackets below each column.

Clonidine was arguably the least effective of the agonists tested; 200 nmol intravitreal clonidine was the only treatment that significantly reduced the differences in negative refractive error and axial elongation, compared to those in PBS controls. However, the reductions induced by this dose of clonidine were quite large, indicating possible toxic effects (n = 10–12; Table 1, Figure 2).

**Figure 3:**
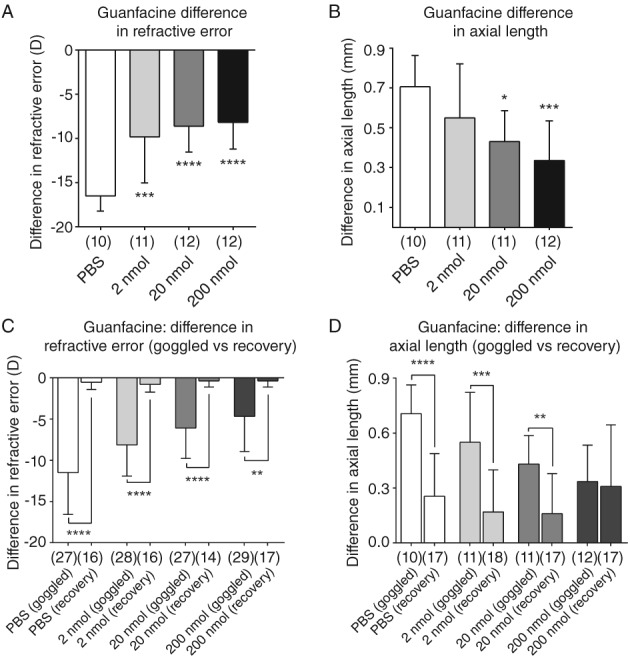
Effect of guanfacine on form‐deprivation myopia in chicks. All concentrations tested significantly inhibited A: the induced negative refractive error, B: but only 20 and 200 nmol resulted in a statistically significant reduction of axial length. C and D: the intraocular difference between goggled eyes and open eyes was reduced significantly after goggles were removed for one week after significant induction of form‐deprivation myopia, with the exception of the axial length of eyes treated with the highest dose (200 nmol) of guanfacine. Data are presented as the means of values ± SD. Statistics: ^****^p < 0.0001, ^***^p < 0.001, ^**^p < 0.01, ^*^p < 0.05; A and B: one‐way analysis of variance + Tukey's post hoc test, and C and D: unpaired t‐test. Sample sizes (n) are denoted in brackets below each column.

All doses of intravitreal guanfacine significantly inhibited the increases in negative refractive error induced by form‐deprivation goggles compared to those in PBS controls, but only 20 and 200 nmol significantly inhibited the associated axial elongation (n = 10–12; Table 1, Figure 3A, B). Eyes treated with 2 and 20 nmol guanfacine completely emmetropised, becoming indistinguishable from control eyes after goggles were removed for one week subsequent to regular drug injections. The refractive error of eyes treated with 200 nmol guanfacine became closer to emmetropia one week after goggle removal, but the excessive axial elongation remained, indicating possible toxic effects on retinal circuitry at the highest dose (n = 14–18; Table 1, Figure 3C, D).

### Effect of α‐adrenoceptor antagonism on form‐deprivation myopia and emmetropisation

**Figure 4:**
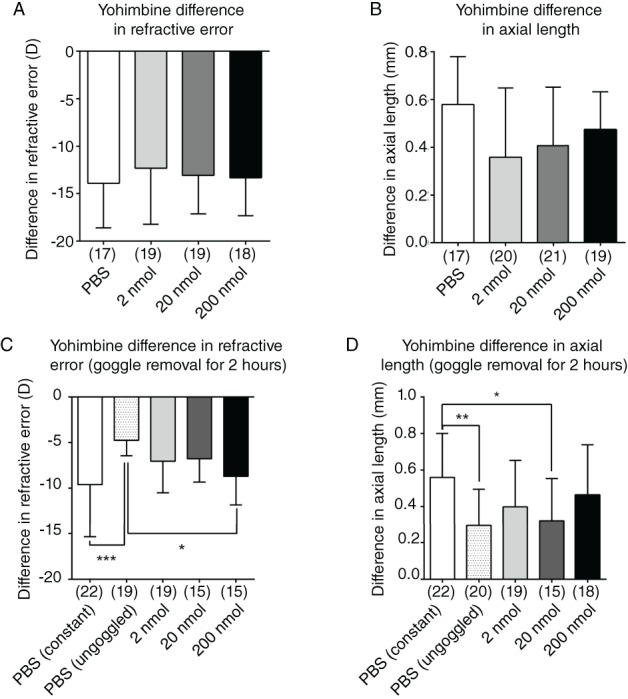
There was no significant effect of yohimbine treatment on the difference in A: refractive error or B: axial length in chick eyes undergoing form‐deprivation myopia. C: There was a small but significant blockade of emmetropisation of refractive error facilitated by goggle removal compared to phosphate‐buffered saline (PBS) controls, D: but there was no significant effect on axial parameters. Data are presented as the means of values ± SD. Statistics: ^***^p < 0.001, ^**^p < 0.01, ^*^p < 0.05; one‐way analysis of variance + Tukey's post hoc test. Sample sizes (n) are denoted in brackets below each column.

Intravitreal injections of yohimbine into goggled eyes had no effect on development of form‐deprivation myopia in any treatment group (n = 17–20; Figure 4A, B). When injected intravitreally before two hours of goggle removal, yohimbine caused a partial blockade of the emmetropisation induced by clear vision, compared to that in PBS controls, but only at the highest dose of 200 nmol.

Axial length may have been affected in parallel with the refractive error (that is, the mean value increased more than in controls), but this effect was not statistically significant. As with previous experiments, there was no blockade of development of form‐deprivation myopia in eyes treated with yohimbine without goggle removal (n = 15–22; Figure 4C, D).

## Discussion

The data presented here provide the first evidence that form‐deprivation myopia is inhibited by high concentrations of brimonidine, clonidine, and guanfacine in the chick. There is also evidence that a high concentration of yohimbine may interfere with the emmetropisation induced by goggle removal. Brimonidine and guanfacine are equally as effective at inhibiting chick form‐deprivation myopia as atropine^5^, ^9^ but the required concentrations still greatly exceed those found to be effective in receptor‐binding and activity assays.^10^, ^14^, ^20^ Thus, the ability of these drugs to modulate changes in eye size cannot be confidently attributed to mechanisms specifically involving α_2_‐adrenoceptors.

Although the effects of intravitreal injections of any drug at very high concentrations cannot be attributed to a specific receptor or system, experiments such as these prompt further investigation of possible downstream signalling mechanisms. For example, it is known that atropine treatment results in a massive increase in the synthesis and release of retinal dopamine^21^ and myopia inhibition by atropine (240 nmol) is blocked by simultaneous injection of a nitric oxide synthase inhibitor (L‐NIO or L‐NMMA; 6 nmol);^9^ nitric oxide and dopamine have been reported to work together in a serial pathway (dopamine acting via nitric oxide) to inhibit myopia.^22^ Myopia inhibition by MT3 is blocked by simultaneous injection of the D_2_‐type antagonist spiperone (5 nmol)^23^ and myopia inhibition by brimonidine may also be dependent on signalling through the dopamine D_2_ receptor (Figure S2).

There is evidence that retinal α_2_‐adrenoceptors may regulate the activity of tyrosine hydroxylase, the rate‐limiting enzyme in dopamine synthesis.^17^ Intravitreal injections of yohimbine (2 nmol) in dark‐adapted rats (both in the dark and at light onset) resulted in increased tyrosine hydroxylase activity, which was partially antagonised by simultaneous intraperitoneal administration of clonidine (7.5 μmol). Clonidine by itself caused a decrease in tyrosine hydroxylase activity when applied at light onset, but had no effect when applied in darkness.

The authors of this study concluded that the effects of clonidine and yohimbine were likely mediated by a combination of α_2_‐adrenoceptor‐ and dopamine‐related mechanisms. The seemingly opposite results reported here (that is, possible activation of dopamine by clonidine, brimonidine, and guanfacine) could be due to a significant species‐difference in the way dopamine affects retinal visual processing in rats (rod‐dominated) and chicks (cone‐dominated). In support of this, there is evidence to suggest that, although increased retinal dopamine inhibits myopia in both chick and rodent (guinea pig) models, it may do so through action at different dopamine receptors (D_2_ in chick^24^ and D_1_ in guinea pig^25^). Clearly, further investigation is required.

Brimonidine has been reported to inhibit lens‐induced myopia in guinea pigs,^13^ which the authors attributed to decreased intraocular pressure. In support of this, a second guinea pig study reports that latanoprost – a prostaglandin analogue used to treat glaucoma – also inhibits form‐deprivation myopia.^26^ Myopic mammals experience a phenomenon called scleral creep, in which the sclera becomes more compliant and prone to deformation in response to myopiagenic stimuli. The highly compliant sclera of myopic mammals could contribute to myopia progression because of elastic stretching, even at normal intraocular pressure. Thus, brimonidine and latanoprost may inhibit guinea pig myopia by reducing physiological intraocular pressure even further and allowing the compliant sclera to ‘relax’.

The chick sclera, unlike that of mammals, does not become more compliant in response to myopiagenic stimuli,^27^ but enlarges mainly by an increase in active scleral growth processes. Accordingly, timolol – a beta‐blocker used to treat glaucoma – is ineffective at inhibiting experimentally induced myopia in the chick, even though it is very effective at decreasing intraocular pressure.^28^ Latanoprost is also ineffective at inhibiting form‐deprivation myopia in the chick, although intravitreal injection of a different prostaglandin, PGF_2α_, is effective.^29^

Interestingly, timolol‐treatment does not inhibit naturally occurring myopia in humans.^30^ Thus, it would be surprising to discover that the mechanism underlying myopia inhibition by α_2_‐adrenoceptor agonists in the form‐deprived chick and form‐deprived and lens‐induced guinea pig models is completely different. Given the significant differences in treatment effectiveness between chicks and guinea pigs, perhaps another explanation should be sought for the anti‐myopia effects of these drugs, independent of intraocular pressure.

Retinal treatment with α_2_‐adrenoceptor agonists results in the activation of neuroprotective mechanisms, such as basic fibroblast growth factor in rat photoreceptors^15^, ^16^ or the extracellular signal‐regulated kinases signalling pathway in chick Müller cells.^12^ Such mechanisms have been shown to be protective against chick form‐deprivation myopia^31^, ^32^ and guinea pig lens‐induced myopia.^33^, ^34^

Although form‐deprivation and lens‐induced models of experimentally induced myopia result in a significant negative refractive error, it is controversial whether they work through the same biological mechanisms.^35^ Form‐deprivation myopia is thought to work primarily through retinal signalling, with no influence from the brain;^36^ severing the optic nerve has no effect on the ability of the eye to develop deprivation myopia^37^ and deprivation of half of the visual field results in myopia only in those areas that correspond to the visually deprived retina.^38^ In contrast, severing of the optic nerve does seem to have an effect on the development of lens‐induced myopia.^39^

Certain drug treatments also reveal a difference between the form‐deprivation and lens‐induced paradigms. 6‐hydroxydopamine and MT3 both inhibit form‐deprivation myopia, but have little or no effect on the development of lens‐induced myopia.^40^, ^41^ Mamba toxin‐1, which is highly selective for mAChR M_1_, inhibits lens‐induced myopia, but has little effect on form‐deprivation myopia in tree shrews.^42^ It is ineffective in chicks because they lack a mAChR M_1_ orthologue.^43^ Atropine has also been reported to be more effective against form‐deprivation myopia than lens‐induced myopia in chicks.^44^

In the present study, the hypothesis that atropine and α_2_‐adrenoceptor agonism may work through different mechanisms was also tested, by combining atropine and guanfacine and measuring the effect on chick form‐deprivation myopia. The differences in refractive error and axial elongation for eyes treated with atropine and guanfacine together were greater than when the drugs were injected separately (Figure S3), suggesting that the effects of the two drugs may be additive.

There are two possible explanations for this result. It could be that the sites and mechanisms of action of the two drugs are the same, and combining the drugs is equivalent to doubling the concentration of one of them, thus causing a stronger inhibitory effect. This is certainly plausible; although the effects of atropine and guanfacine seem to plateau at concentrations ≥ 20 nmol in these chick strains; concentrations > 10 mmol/L were not tested. Second, atropine and guanfacine, at high concentrations, might actually affect different myopia‐inhibiting pathways in the eye.

These experiments are similar in theory to a previous study investigating the effects of combination of atropine and apomorphine, which did not find an additive effect.^45^ That the effects of atropine and guanfacine were additive, while those of atropine and dopamine were not, is consistent with the idea of multiple emmetropisation pathways in the eye, and/or the possibility of multiple sites of action for growth‐regulating processes. This is an interesting avenue for future investigations.

## Conclusion

The results from these studies are relevant to a common misconception in the myopia research field: namely, that the results from high‐concentration drug treatments injected into the eye can be attributed to a specific receptor or system. The data presented here are far from proving that α‐adrenoceptors are valid target receptors for anti‐myopia therapies, but they provide yet another class of drugs that can inhibit myopia at high concentration. In doing so, they support the argument that the field should be much more circumspect in attributing the effects of a high‐concentration drug to a specific receptor or visual system.

## Supporting information


**Figure S1.** Refractive error (left axis) and axial length (right axis) data for ungoggled control eyes from dose‐response experiments. No drug treatment significantly altered the growth of the control eyes. Statistical data (p‐ and F‐values) are reported at the bottom of the columns (one‐way ANOVA + Tukey's post‐hoc). Data are presented as the means of values (refractive error or axial length) ± SD. Sample sizes (n) are denoted in brackets below each column.
**Figure S2.** The effect of brimonidine (20 nmol), spiperone (4 nmol) and their combination on form‐deprivation myopia in chicks. Addition of spiperone with brimonidine resulted in the blockade of inhibition of form‐deprivation myopia by brimonidine. DMSO: dimethyl sulfoxide. ***p < 0.001, *p < 0.05 (one‐way ANOVA + Tukey's post‐hoc). Data are presented as the means of the difference in values for the experimental eye minus those for the control eye ± SD. Sample sizes (n) are denoted in brackets below each column.
**Figure S3.** The effect of guanfacine (20, 200 nmol), atropine (80, 200 nmol) and guanfacine (200 nmol) + atropine (200 nmol) on form‐deprivation myopia in chicks. The combined effect of guanfacine and atropine resulted in a greater inhibition of form‐deprivation myopia than guanfacine or atropine alone. PBS: phosphate‐buffered saline. In comparison to guanfacine + atropine; ^****^p < 0.0001, ^***^p < 0.001, ^*^p < 0.05; one‐way ANOVA + Dunnet's post hoc test. Data are presented as the means of the difference in values for the experimental eye minus those for the control eye ± SD. Sample sizes (n) are denoted in brackets below each column.
