# Supplementary material for: Alpha2‐adrenoceptor agonists inhibit form‐deprivation myopia in the chick
Source: Clin Exp Optom. 2019 Jan 30;102(4):418–25. doi: 10.1111/cxo.12871 (PMC6617789; doi:10.1111/cxo.12871)
Supplement: Supplementary file 1 — Figure S1. Refractive error (left axis) and axial length (right axis) data for ungoggled control eyes from dose‐response experiments. No drug treatment significantly altered the growth of the control eyes. Statistical data (p‐ and F‐values) are reported at the bottom of the columns (one‐way ANOVA + Tukey's post‐hoc). Data are presented as the means of values (refractive error or axial length) ± SD. Sample sizes (n) are denoted in brackets below each column. Figure S2. The effect of brimonidine (20 nmol), spiperone (4 nmol) and their combination on form‐deprivation myopia in chicks. Addition of spiperone with brimonidine resulted in the blockade of inhibition of form‐deprivation myopia by brimonidine. DMSO: dimethyl sulfoxide. ***p < 0.001, *p < 0.05 (one‐way ANOVA + Tukey's post‐hoc). Data are presented as the means of the difference in values for the experimental eye minus those for the control eye ± SD. Sample sizes (n) are denoted in brackets below each column. Figure S3. The effect of guanfacine (20, 200 nmol), atropine (80, 200 nmol) and guanfacine (200 nmol) + atropine (200 nmol) on form‐deprivation myopia in chicks. The combined effect of guanfacine and atropine resulted in a greater inhibition of form‐deprivation myopia than guanfacine or atropine alone. PBS: phosphate‐buffered saline. In comparison to guanfacine + atropine; ****p < 0.0001, ***p < 0.001, *p < 0.05; one‐way ANOVA + Dunnet's post hoc test. Data are presented as the means of the difference in values for the experimental eye minus those for the control eye ± SD. Sample sizes (n) are denoted in brackets below each column. [file CXO-102-418-s001.docx]

**Supplementary Figure S1**: Refractive error (left axis) and axial length (right axis) data for ungoggled control eyes from dose-response experiments. No drug treatment significantly altered the growth of the control eyes. Statistical data (p- and F-values) are reported at the bottom of the columns (One-Way ANOVA + Tukey’s post-hoc). Data are represented as the means of values (refractive error or axial length) ± SD. Sample sizes (n) are denoted in brackets below each column.





**Supplementary Figure S2**: The effect of brimonidine (20 nmoles), spiperone (4 nmoles) and their combination on form-deprivation myopia in chicks. Addition of spiperone with brimonidine resulted in the blockade of inhibition of form-deprivation myopia by brimonidine. *Abbreviations:* DMSO: dimethyl sulfoxide. *Statistics*: ***p < 0.001, *p < 0.05 (One-Way ANOVA + Tukey’s post-hoc). Data are represented as the means of the difference in values for the experimental eye minus those for the control eye ± SD. Sample sizes (n) are denoted in brackets below each column.





**Supplementary Figure S3**: The effect of guanfacine (20, 200 nmoles), atropine (80, 200 nmoles) and guanfacine (200 nmoles) + atropine (200 nmoles) on form-deprivation myopia in chicks. The combined effect of guanfacine and atropine resulted in a greater inhibition of form-deprivation myopia than guanfacine or atropine alone. *Abbreviations:* PBS: phosphate-buffered saline. *Statistics*: In comparison to guanfacine + atropine; ^****^p < 0.0001, ^***^p < 0.001, ^*^ p < 0.05; One-Way ANOVA + Dunnet’s post-hoc. Data are represented as the means of the difference in values for the experimental eye minus those for the control eye ± SD. Sample sizes (n) are denoted in brackets below each column.
